# Supplementary material for: Bone Marrow Endothelial Cells Increase Prostate Cancer Cell Apoptosis in 3D Triculture Model of Reactive Stroma
Source: Biology (Basel). 2022 Aug 26;11(9):1271. doi: 10.3390/biology11091271 (PMC9495890; doi:10.3390/biology11091271)
Supplement: Supplementary file 1 [file biology-11-01271-s001.zip › biology-1803022-supplementary.pdf]

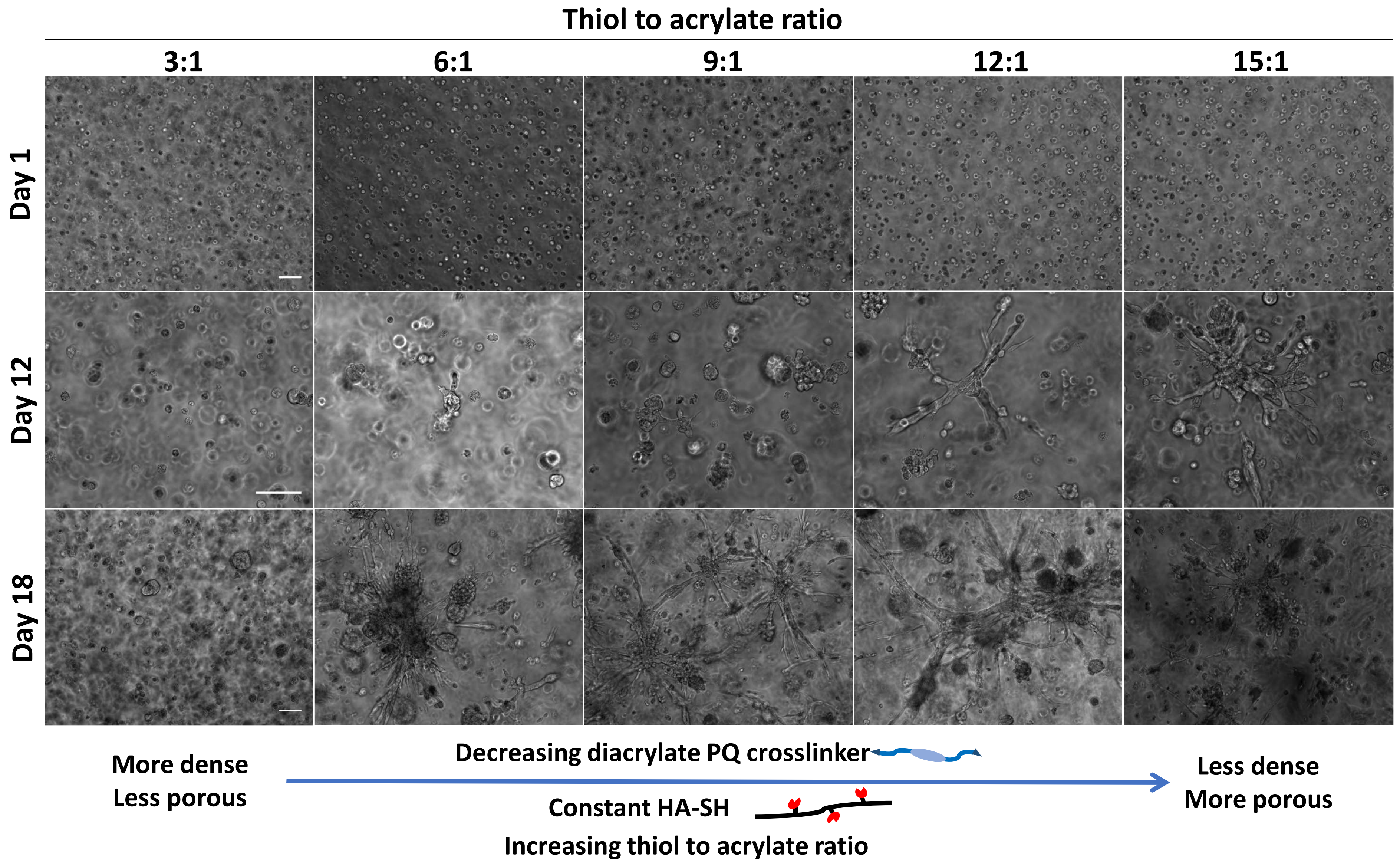

Figure S1. Lower hydrogel crosslinking density increases cellular invasion and organization.

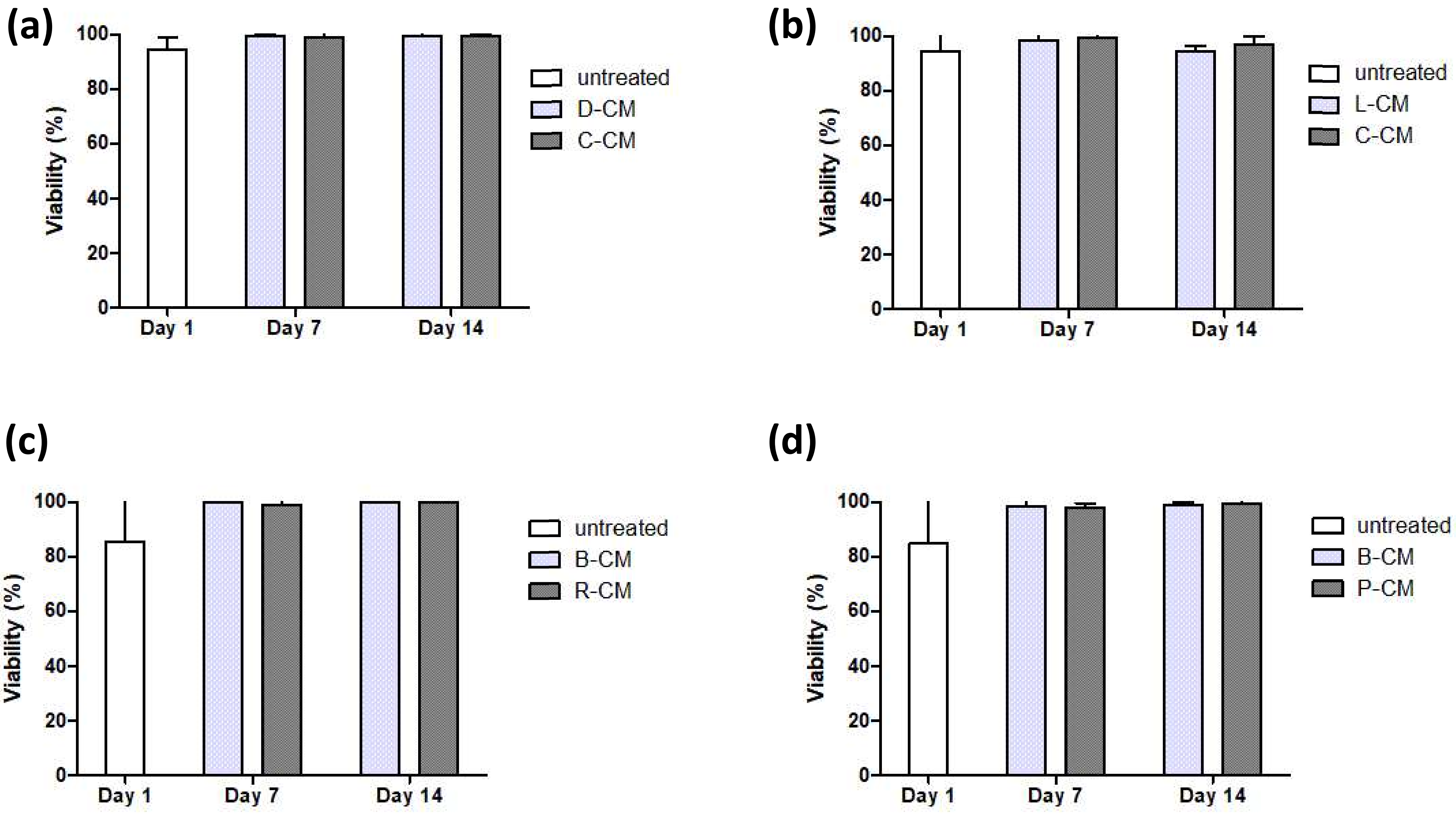

Figure S2. HMEC-1- and HULEC-5a-conditioned media do not affect C4-2B viability, and BMEC-conditioned medium does not affect 22Rv1 or PC3 viability.

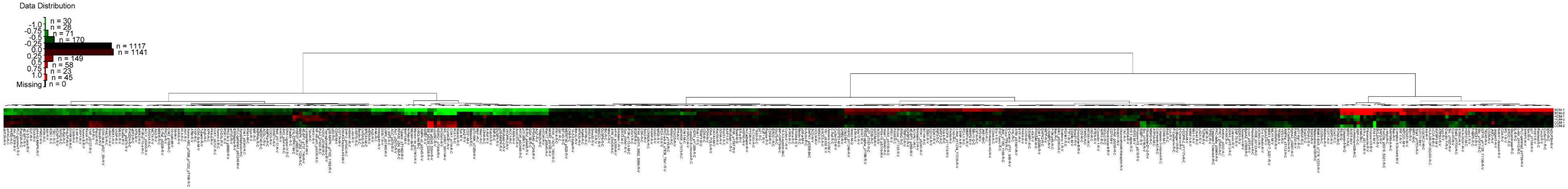

Figure S3. Full results of RPPA analysis.

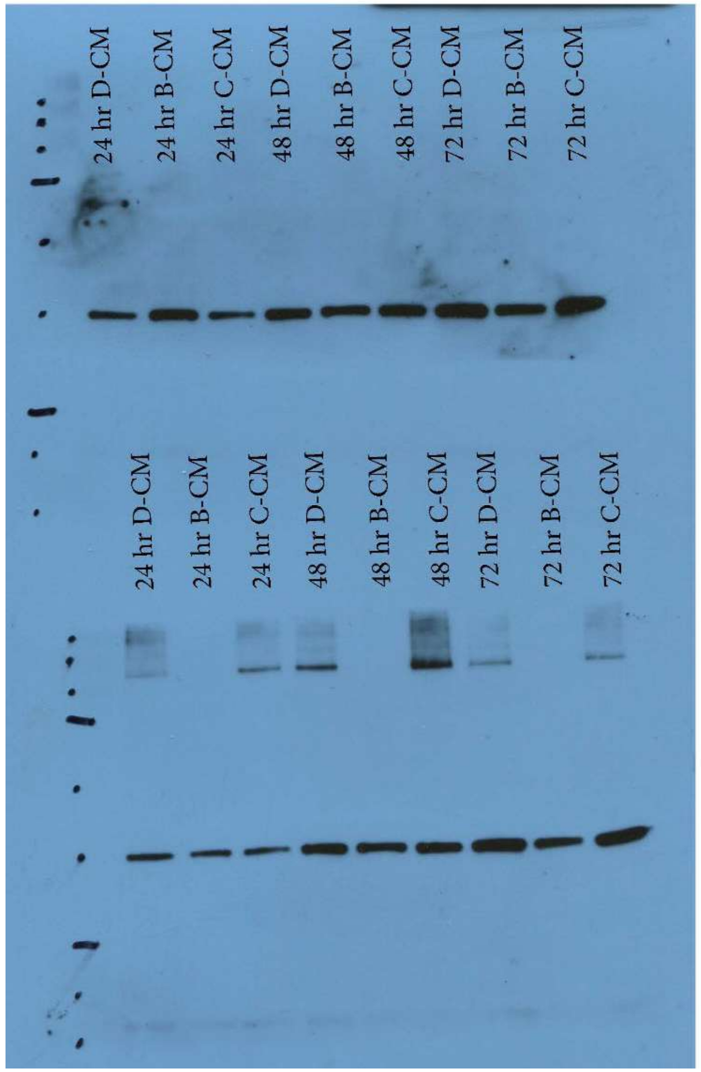

GAPDH in Figure 6b

pRb in Figure 6c

GAPDH in Figure 6c

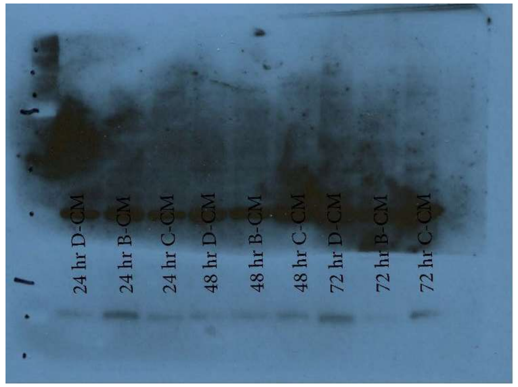

Smac in Figure 6b

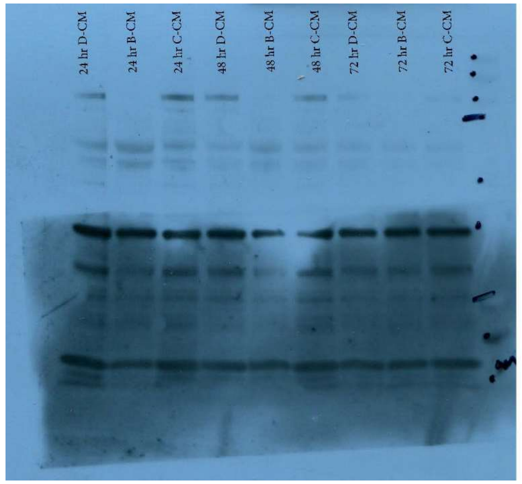

FOXM1 in Figure 6d

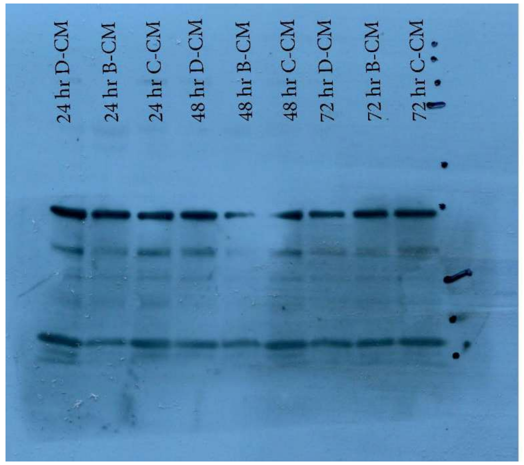

GAPDH in Figure 6d

Figure S4. Original images of full western blot.
